# Ecological Effect Assessment of Low-Carbon City Construction in China

**DOI:** 10.3390/ijerph192114467

**Published:** 2022-11-04

**Authors:** Juan Yin, Jin Guo

**Affiliations:** 1 College of Intelligent Science and Control Engineering, Jinling Institute of Technology, Nanjing 211169, China; 2 School of Business, Nanjing Normal University, Nanjing 210023, China

**Keywords:** low-carbon pilot policy, urban green development, PSM-DID model

## Abstract

This paper takes the second batch of low-carbon pilot cities in China as the research object and selects the Urban Health Ecological Index to measure the green development level of cities, aiming to explore and evaluate the theoretical mechanism and policy effect of low-carbon pilot projects to promote the coordinated development of urban economy, society and the environment. The research conclusions show that: ① The low-carbon city pilot project is conducive to support the pilot cities to build a low-carbon industrial system, advocate a low-carbon lifestyle, establish a low-carbon evaluation system, and then play a positive role in promoting the green development level of the city; ② By applying the Propensity Score Matching–Difference in Differences (PSM-DID) model, the empirical analysis finds that after the implementation of the pilot policy, the green development level of low-carbon pilot cities has been significantly improved, and this conclusion is still stable in the parallel trend test, counterfactual test and sample expansion test; ③ In terms of regional heterogeneity, the low-carbon pilot projects have a more significant policy effect on promoting the green development of provincial capitals and eastern cities. Strict administrative supervision in provincial capitals and good economic foundations in eastern cities have had a positive moderating effect on the policy effect of low-carbon pilot projects. Finally, this paper discusses how to realize the ecological effects of low-carbon city pilot projects and put forward some relevant policy suggestions.

## 1. Introduction

Since the first industrial revolution and especially after World War II, the process of global urbanization advanced rapidly [1]. According to World Bank statistics, 4.36 billion people lived in cities in 2020, accounting for 56% of the world’s population. Urbanization rates are even higher in upper-middle- and high-income economies, at 68% and 82%, respectively. Cities have become important platforms for people to work and live. Predictably, the importance of cities in economic development will become more prominent as the global division of labor deepens and as low- and middle-income economies develop [2,3].

However, the influx of large numbers of people into cities also has various adverse consequences, especially in the areas of resource scarcity and environmental pollution [4,5]. These urban diseases continue to corrode cities’ delicate ecology and pose a serious threat to sustainable urban development. Urban ecology is deteriorating, and population migration is even experiencing a wave of anti-urbanization in many countries. Against this background, governments from various countries have been exploring how to restore the ecology of cities and construct urban spaces in a way that harmonizes social, economic and natural development. Countries around the world are actively exploring low-carbon development to cope with this problem [6,7]. Numerous studies have analyzed low-carbon governance at the local level from different perspectives in different cities [8,9,10].

Following reform and opening up, China has undergone the fastest and most extensive urbanization process in the world. However, the original intention of urbanization in China was not to build habitable cities, but to serve its industrial development by releasing demographic dividends. The Chinese government has adopted the *Hukou* system, which only allows rural migrants to work rather than live in the city; this has eased some of the shortage of urban public resources [11]. However, with the progress of *Hukou* reform and the gradual collapse of registration discrimination, China’s urban population has also witnessed an explosive growth, constantly hitting the urban ecological red line. A typical incident is the concentrated outbreak of haze pollution in several Chinese cities in 2013.

The Chinese government has started to address urban diseases as the country moves into a post-industrial phase. The 2010 Shanghai World Expo’s catchphrase, “Better city, Better life”, served as a rallying cry for the Chinese city government to alter the path of urban management. In 2010, the Chinese government also launched a pilot program to build low-carbon cities. The plan offers new ideas to actively explore the rapid development phase of China’s industrialization and urbanization, which can both stimulate the economy and also tackle environmental degradation and promote green development in cities. In July 2010, the National Development and Reform Commission (NDRC) launched the first batch of low-carbon pilot projects and identified five provinces (Guangdong, Liaoning, Hubei, Shanxi, Yunnan) and eight cities (Tianjin, Chongqing, Shenzhen, Xiamen, Hangzhou, Nanchang, Guiyang, Baoding) as pilot regions. The plan requires pilot regions to calculate and determine their own total greenhouse gas emission control targets, study and formulate plans for the allocation of greenhouse gas emission targets, establish a local carbon emission trading supervision and registration system, cultivate and build trading platforms, and build a pilot support system for carbon emission trading. Obviously, the main purpose of implementing this policy is to encourage the development of green technologies, improve the low-carbon industrial system, promote the optimization and upgrading of the urban industrial structure and the clean transformation of the energy consumption structure, improve energy efficiency, and ultimately achieve green and ecological development of the city.

**Figure 1:**
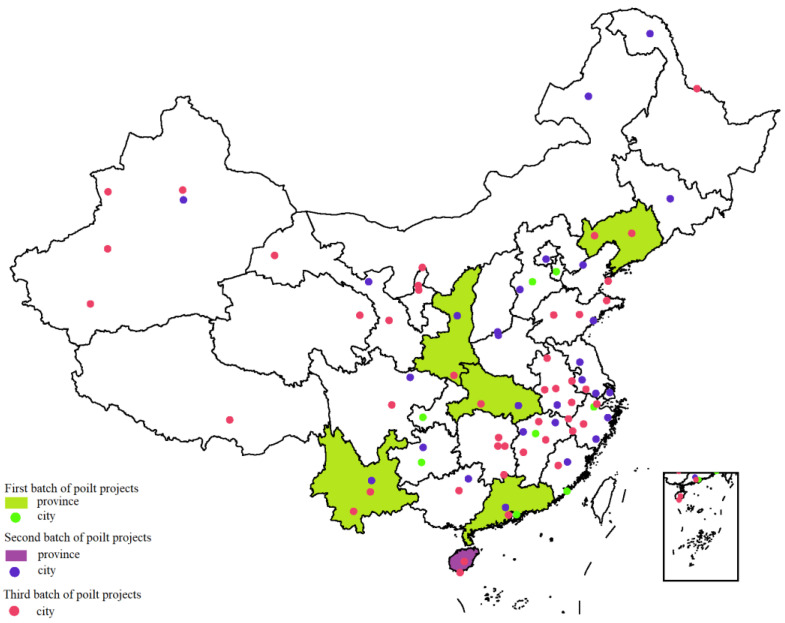
Spatial distribution of three batches of low-carbon pilot regions in China.

After the initial achievements in the construction of the first batch of low-carbon pilot projects, the NDRC continued to announce the second and third batches of low-carbon pilot projects in November 2012 and January 2017, respectively. So far, China’s inland areas have formed a low-carbon development pattern in which 31 provincial-level administrative units have at least one pilot region. Figure 1 shows the spatial distribution of the three batches of low-carbon pilot areas. It can be found that in the second and third batch of low-carbon pilot projects, prefecture-level cities and county-level cities under the jurisdiction of provincial administration have become the main targets for China to promote low-carbon pilot construction. Compared with provinces, prefecture-level cities and county-level cities can more fully combine urban characteristics to formulate targeted low-carbon city construction plans. This trend indicates that China’s low-carbon pilot projects are further “sinking”, with increasing attention being paid to the implementation of top-level design policies.

Faced with the dilemma of sustainable development in traditional cities, low-carbon cities, as a novel mode of urban development, offer a viable solution to the contradiction between urban development, resource conservation, and environmental protection. At the current stage, the ecological level of Chinese cities has significantly improved, and many achievements have been made in urban green development. This motivates us to consider whether there is a causal link between the improvement of the urban ecological environment and the implementation of low-carbon urban pilot projects. Answering this question is of great practical importance for further exploring viable paths for urban green development and improving the quality of the urban environment. Therefore, in this paper we focus on the following two issues:

i. Will low-carbon city pilot policies help raise the level of green urban development?
ii. What are the differences in the ecological impact of low-carbon city pilot policies in different cities? What accounts for this discrepancy?

The possible contributions of this paper encompass three aspects:

First, instead of directly using energy conservation or carbon emission reduction indicators as proxy variables, we chose the Urban Health Ecological Index to measure the level of green development in each city. The purpose of this study is not to assess the energy-saving or carbon-reduction effects of pilot policies in low-carbon cities, but rather to assess the combined impact of pilot policies on the coordinated development of the city’s economy, society and environment. This is a crucial dialectical logic: the more critical purpose of low-carbon city pilot projects is to achieve the “win–win” of economic development and environmental protection. If the goal of energy conservation or carbon reduction is achieved at the expense of economic development, this would be a departure from the original intent of the low-carbon city pilot policy.

Second, we take the second group of relatively mature low-carbon pilot cities as the research object, and collect and carefully study their low-carbon development plans and implementation schemes. We uncover how a city works when it is chosen to become a low-carbon center. We point out that building low-carbon industrial systems, advocating low-carbon lifestyles, and establishing low-carbon assessment constraints are the main channels for low-carbon pilot cities to promote urban green development.

Third, we apply the DID model to scientifically assess the ecological impact of low-carbon pilot projects on the basis of alleviating the endogeneity issues. We also explore the influence of administrative supervision and economic bases on low-carbon pilot projects, and find that the positive impact of low-carbon pilot projects on promoting urban green development is stronger in provincial capitals with stricter administrative supervision and in eastern cities with a better economic basis.

The paper is structured as follows. Section 2 provides a review of the literature. Section 3 describes the theoretical mechanism. Section 4 presents the research design and data. Section 5 is devoted to empirical results, robustness tests, and heterogeneity analysis. Section 6 summarizes the conclusions and provides policy recommendations.

## 2. Literature Review

The low-carbon economy was first proposed by Kinzig and Kammen [12] as a novel urban development mode. It is conducive to achieving the three goals of urban development, resource conservation, and environmental protection [13,14]. The low-carbon city aims to implement a low-carbon economy and build a green economic development model within the city. The green development of a city refers to the coordinated development of the urban economy, society, and environment after considering environmental constraints [15,16,17]. Compared with the low-carbon economy with the direct purpose of energy conservation and emission reduction, the green development of cities emphasizes the symbiosis of economy, society, and the natural environment [18,19]. Low-carbon construction in cities has a natural connection to urban green development. Realizing low-carbon development is a systematic project to develop the green economy [20,21,22]. As a result, the construction of low-carbon pilot cities as an opportunity to promote green urban development has also been hotly debated by scholars. Fu and Zhang [23] pointed out that among all the urban concepts of sustainable development, low-carbon cities have been recognized by the government in China, and low-carbon pilot cities are spreading across the country.

After combing the relevant literature, this paper argues that the existing research is mainly divided into three branches: The first focuses on the construction paths of low-carbon provinces and cities. For example, Yang and Li [24] analyzed the reasons for China’s choice of a low-carbon path and the development of low-carbon cities from the three aspects of resource-saving and environmentally friendly industrial structures, production modes, and lifestyles, and put forward specific requirements for the construction of low-carbon cities. Zhang et al. [25] applied Kaya identity to analyze the influencing factors and driving forces of carbon emission characteristics of China’s low-carbon pilot provinces and proposed different countermeasures for different regions. Yang et al. [26] selected the core indicators of economy and carbon emissions for cluster analysis and divided low-carbon cities into four types—leading type, development type, late type, and exploration type—and combined with each type, gave a specific construction path for a low-carbon city. Yang et al. [27] proposed a new carbon tax–constrained urban logistics distribution network planning model for the city’s logistics distribution system, which is a department with heavy energy consumption and serious pollutant emissions. In the case of Beijing, they noted that carbon tax constraints could reduce the city’s carbon dioxide emissions by about 54.5%.

The second branch of literature, based on a historical perspective, assesses the low-carbon development performance of pilot provinces and cities using either a single index or a comprehensive index evaluation method. In China, some ministries and commissions have set a series of index systems relevant to the concept of a low-carbon city, such as the Green Development Index System (NDRC, 2016) and the Evaluation Target System of Ecological Civilization Construction (NDRC, 2016) (the NDRC issued the “Notice on Publishing Green Development Index System and Evaluation Target System of Ecological Civilization Construction” in 2016). Academics have conducted extensive research focusing on the evaluation systems for low-carbon cities. The primary indicators selected by scholars in constructing the index system are mainly related to energy, economy, society, and environment [28,29,30,31]. Some evaluation index systems for low-carbon cities were constructed based on the five dimensions of “driving force–pressure–state–influence–response”, which measures and compares the low-carbon development levels of those cities from pilot areas before and after being pilot areas [32,33]. Guo et al. [34] established the evaluation index system of China’s low-carbon cities from the two aspects of driving force and resistance, and evaluated and classified the urban carrying capacity of Wuhan urban agglomeration using principal component analysis and cluster analysis. The evolutionary trajectory of the low-carbon competition in most cities in the Wuhan urban agglomeration showed an upward trend from 2007 to 2011, but some cities were U-shaped or vibrating, the study concluded. It is important to note that these studies assess the policy impact of low-carbon pilot projects by comparing the differences before and after the implementation of low-carbon pilot policies in pilot regions. The research method belongs to the category of “single difference analysis”. The net effect of low-carbon pilot policies cannot be accurately assessed because the effect of time trend factors cannot be eliminated.

In view of the shortcomings in research methods of the second group of literature, the third branch of literature introduces the concept of quasi-natural experiments, constructs a Difference in Difference (DID) model, and comprehensively evaluates the policy effect of low-carbon pilot projects by comparing the differences between pilot samples and non-pilot samples before and after the pilot project. For example, Huo et al. [35] adopted the DID model approach to study the carbon emission reduction effect of the low-carbon city pilot policy, finding that it can significantly reduce the per capita carbon emissions; the longer the pilot time, the more obvious the carbon emission reduction effect. Using the DID model, Lin et al. [36] and Yan et al. [37] empirically found that the low-carbon city pilot policy largely reduced API, PM2.5, and other air pollution indexes and improved urban air quality. The low-carbon city pilot policy has significantly improved urban ecological efficiency [38]. Meanwhile, the low-carbon city pilot policy can improve the green total factor productivity of pilot cities through the effects of technological innovation, industrial structure, resource allocation, energy intensity, and carbon sequestration [39,40]. In view of the difficulty that the experimental group and the control group in the DID model have difficulty meeting the premised hypothesis of a common trend, some scholars use the quasi-natural experiment method of Propensity Score Matching-Difference in Difference (PSM-DID) to evaluate the effectiveness of the low-carbon pilot city, examining to what extent and through which mechanisms the scheme contributes to the ecological performance of Chinese cities [41,42,43]. Fu et al. [44] used the Malmquist–Luenberger productivity index in DEA and the quasi-experimental method of PSM-DID to evaluate the impacts of the low-carbon city pilot scheme in China. The conclusions of the study still support a significant and sustained role for pilot policies in driving down the intensity of urban carbon emissions. However, the top-level design of the central government is often distorted by the poor implementation of local officials, resulting in inefficient or even ineffective policies [45]. At the level of policy planning and implementation, there are such defects as scattered goal setting, mismatching of the main body’s rights and responsibilities, and overly broad policy scopes [46]. In view of these problems, Khanna et al. [46] and Song et al. [47] pointed out that China’s low-carbon city pilot projects in the future need to further emphasize the “city-based policy”, clarify the planning objectives of each pilot city and building a clear and authoritative institutional support and restraint mechanism.

## 3. Theoretical Mechanism

Because the first batch of low-carbon pilot areas are mainly provincial administrative units and only involve eight cities (including Tianjin and Chongqing), the sample size is relatively small. Although the third batch of low-carbon pilot cities has a large sample size, the timelines is relatively short, and the policy effect may not be fully presented. In view of the policy lag, we select the second batch of low-carbon pilot cities, with a certain sample size and relatively mature development, to be the subject of this paper and conduct theoretical mechanism analysis and policy effect evaluation.

**Figure 2:**
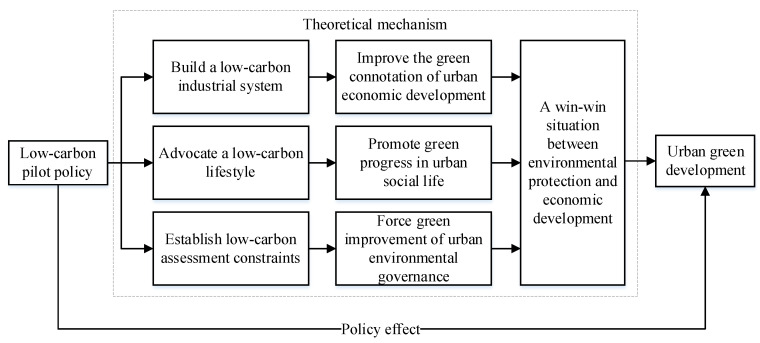
Theoretical mechanism for low-carbon pilot policies to promote green development.

Compared with non-pilot cities, approved low-carbon pilot cities will enjoy a wide range of national policy priorities, such as environmental protection, circular economy, energy conservation, emission reduction, new energy industries, and construction of green and low-carbon towns. Incentives at the policy level will encourage pilot cities to actively explore ways to achieve coordinated economic, social, and environmental development. At the same time, low-carbon development performance will also face more restrictive regulations and evaluation after a city is included in the pilot project. We collected and collated the low-carbon development plans and implementation schemes officially announced by the second batch of low-carbon pilot cities and extracted statements that reflect the key work of the low-carbon pilot construction in each city. In view of the fact that urban green development not only focuses on energy conservation and emission reduction, but also emphasizes the coordinated development of urban economy–society–environment after considering environmental constraints [48], this paper identifies the theoretical mechanism of the low-carbon pilot policy to promote urban green development from three aspects (shown in Figure 2):

i. Building a low-carbon industrial system to improve the green connotation of urban economic development;
ii. Advocating a low-carbon lifestyle to promote green progress in urban social life;
iii. Establishing low-carbon assessment constraints to force green improvement of urban environmental governance.

(1) Building a low-carbon industrial system to improve the green connotation of urban economic development. Industry is the backbone of a city’s economic development, and the negative externality of industrial production on the environment directly affects the green quality of urban economic development. The government can adjust the industrial structure by implementing supporting industrial policies, so as to improve the ecological environment of the city [49,50]. Under the guidance of the government’s low-carbon policy, low-carbon pilot cities will promote the transformation and upgrading of industrial structures in the direction of high-tech content [51], low pollution emissions, and low-energy consumption; policies will gradually eliminate high-pollution and low-efficiency industries, cultivate and support strategic emerging industries, and finally establish a low-carbon industrial system characterized by low-pollution, low-emission, and low-energy consumption. Zhu et al. [52] pointed out that industrial structure adjustment is the key measure to achieve the goal of green economic development. Under the guidance of low-carbon city pilot policies, the traditional pollution-intensive industries will be phased out, and low-carbon industries consistent with the city’s advantages will be developed, such as low-carbon agriculture, new energy industries, and emerging industries represented by green and low-carbon technologies [53].

Based on the analysis of the low-carbon development planning and implementation schemes of the second batch of pilot cities, all the pilot cities have very detailed construction ideas on how to build a low-carbon industrial system. For example, Shijiazhuang, a prefecture-level city in Hebei Province, positioned the construction of low-carbon industrial system first of the seven key tasks in constructing a low-carbon city. It also planned the construction of low-carbon industries using four aspects: cultivating emerging industries, transforming high-carbon industries, eliminating backward industries, and building modern service industries. The implementation scheme of the pilot low-carbon urban construction project in Jincheng, a prefecture-level city in Shanxi province, fully summarizes the challenges facing the construction of a low-carbon industrial system. It notes that the secondary industries in the industrial system of Jincheng are extensive, about 17% above the national average, while the development of the tertiary industries is lagging behind, at about 11% below the national average. In this context, Jincheng designed a path for the construction of a low-carbon industrial system, such as the implementation of comprehensive control, transformation and upgrading of traditional industries, the construction of a diversified low-carbon industrial support model, and the improvement of resource utilization efficiency and industrial energy efficiency.

To sum up, we propose the first theoretical mechanism for low-carbon city pilot projects as the promotion of urban green development.

Theoretical Mechanism 1. Low-carbon city pilot projects are conducive to supporting pilot cities to build low-carbon industrial systems, thereby enhancing the green connotation of urban economic development.

(2) Advocating a low-carbon lifestyle to promote green progress in urban social life. In addition to industrial production, carbon emissions from residents’ living areas are also a significant source of urban carbon emissions. In the rapid development of urbanization in China, the task of reducing carbon emissions in the residential sector has become increasingly difficult. The research by Liu et al. [54] found that the carbon emissions from household activities accounted for 42.17–49.12% of China’s total carbon emissions from 1992 to 2007. Calculations by Wang et al. [55] showed that the total carbon emissions of China’s households increased from 861.41 million tons in 2006 to 1418.24 million tons in 2010, with an average annual growth rate of 13.27%. Therefore, advocating a low-carbon lifestyle is an inevitable path for developing low-carbon cities. It is worth noting that the cultivation of a low-carbon lifestyle for residents is not only reflected in energy saving and emission reduction but is also an important means to alleviate the contradiction between social life and environmental protection and promote green progress in urban life.

As far as the second batch of low-carbon pilot cities is concerned, the implementation of the construction of low-carbon pilot cities in Qinhuangdao, another prefecture-level city in Hebei Province, has seen suggestions regarding innovation in low-carbon lifestyle from the aspects of promoting low-carbon consumption, developing low-carbon tourism, strengthening the treatment of urban waste resources, and raising the awareness of people. It should be pointed out that there are numerous carbon emission channels in residential life, but the carbon emissions in transportation account for the highest proportion, which is the top priority to ensure emission reduction. The city of Jilin proposed an urbanization and transportation decarbonization path, with specific measures that include prioritizing public transportation, encouraging people to change the way they travel, implementing higher emission standards for buses and private cars, and encouraging the development of new energy vehicles. In view of the current situation of a large floating population and high carbon emissions from transportation, the city of Suzhou conducted short-term, medium-term, medium-long-term and long-term quantitative assessments of the target value of low-carbon consumption in the compiled Suzhou Low-carbon Development Plan. For example, the bus-sharing rate increased from 22.6% in 2010 to 26% in 2015, 32% in 2020, 37% in 2025, and 41% in 2030. Energy conservation and emission reduction in the construction sector are also important tasks for the construction of low-carbon cities. According to the implementation schemes of Wuhan, a prefecture-level city in Hubei Province, a policy system, standard system, and management system for low-carbon buildings should be formulated to promote the construction of national renewable energy building application cities and green building demonstration zones.

To sum up, we propose the second theoretical mechanism for low-carbon city pilot projects as the promotion of urban green development.

Theoretical Mechanism 2. Low-carbon city pilot projects are conducive to supporting pilot cities to advocate for a low-carbon lifestyle, thereby promoting green advances in urban social life.

(3) Establishing low-carbon assessment constraints to force green improvements in urban environmental governance. In view of the public product attributes of environmental resources, existing studies have basically affirmed the important role of environmental regulation in promoting the internalization of environmental pollution costs [56,57,58]. While low-carbon pilot policies cannot be considered a regular environmental regulatory tool, low-carbon pilot cities that have been approved by the state often face stricter carbon emission constraints. The low-carbon assessment constraint will encourage cities to strengthen supervision and assessment of energy conservation, emission reduction, environmental governance, and ecological restoration and produce environmental governance effects similar to environmental regulations, thereby promoting urban green development.

The implementation schemes of Wuhan’s low-carbon city pilot work have sought to incorporate the low-carbon pilot work into the municipal performance management objective evaluation system; implement a strict performance management objective evaluation system; establish an evaluation index system for low-carbon urban areas, low-carbon parks, low-carbon enterprises, and low-carbon communities; and conduct regular inspection and assessment. The 13th Five-Year Plan for low-carbon development in Wuhan further emphasized that the carbon emission reduction tasks assigned by the state should be decomposed and implemented in relevant key emission enterprises; annual assessment should be conducted, reward and punishment measures should be improved, and strict accountability should be implemented. As a city with a high concentration of manufacturing enterprises, Guangzhou, the capital of Guangdong province, has paid special attention to strengthening the management of carbon emissions by key enterprises under the 13th Five-Year Plan for Energy Conservation and Carbon Reclamation. It lists industrial enterprises with an annual energy consumption of 5000 tons of standard coal as key energy consuming enterprises and regularly checks their energy utilization conditions and pollutant emissions. In addition to regulating and restricting the production behavior of enterprises, many low-carbon pilot cities have proposed measures to enhance the urban carbon sink and pollutant purification capacity. For example, the city of Guilin proposed to improve the city’s carbon sink capacity and livable comfort by increasing forest coverage.

To sum up, we propose the third theoretical mechanism for low-carbon city pilot projects as the promotion of urban green development.

Theoretical Mechanism 3. Low-carbon city pilot projects are conducive to supporting pilot cities to establish low-carbon assessments, thereby improving the governance system for urban green development.

## 4. Research Design

### 4.1. Model Settings

In this study, the low-carbon pilot policy is regarded as a quasi-natural experiment [59,60]. A quasi-natural experiment is a method of social science research. Compared to real experimental studies, it employs certain manipulation procedures, exploits natural scenarios, and has flexible control over the experimental subjects. Unlike real experimental design, subjects are not randomly assigned to the experimental and control groups. Considering the lag of policy, this paper selects the second batch of low-carbon pilot cities with a certain scale and relatively mature development as the research objects. After eliminating the first batch of low-carbon pilot cities and the cities under the jurisdiction of the first and second batch of pilot provinces, the second batch of low-carbon pilot cities are taken as the experimental group and other non-pilot cities as the control group. We use the Difference in Difference (DID) model, which is commonly used in the field of policy evaluation to test the policy effect of low-carbon pilot policy in promoting urban green development. On this basis, the econometric model, as shown in Equation (1), is constructed:

(1) $$Green_{it}=\beta_{1}City_{i}+\beta_{2}Policy_{t}+\beta_{3}City_{i}\times Policy_{t}+\lambda X_{it}+\nu_{i}+\mu_{t}+\varepsilon_{it}$$

where $Green_{it}$ represents the green development level of city *i* in period *t*; $City_{i}$ is a city dummy variable that takes on 1 if the city is one of the low-carbon pilot cities in 2012, and 0 otherwise; $Policy_{t}$ is the break point of policy implementation. Since the second batch of low-carbon pilot cities was identified in November 2012, this paper sets 2013 as the break point for policy implementation and assigns a value of 1 to 2013 and after; otherwise, the value is 0; $X_{it}$ is a group of control variables, including the level of economic development, industrial structure, and the scale of foreign investment $\nu_{i}$ and $\mu_{t}$ are the city fixed effect and time fixed effect, respectively; and $\varepsilon_{it}$ is the random disturbance term. In order to reduce heteroscedasticity and nonlinear problems as much as possible, the continuous variables are included in the model in the form of logarithms. When the interactive term City×Policy is 1, it denotes the scenario of the experimental group cities after the implementation of the low-carbon pilot policy. Therefore, its regression coefficient $\beta_{3}$ reflects the impact of low-carbon pilot policies on urban green development. The regression result of $\beta_{3}$ is expected to be significantly positive, that is, the low-carbon pilot can significantly promote urban green development.

Furthermore, in order to reduce the bias of regression results caused by the failure of the experimental group and the control group to meet the common trend hypothesis as far as possible, this paper uses Propensity Score Matching—Difference in Difference (PSM-DID) to fit the coefficients in Equation (1).

### 4.2. Variable Definitions

This article selects the Urban Health Ecological Index from the “China Eco-City Construction and Development Report” from 2008 to 2015 to measure the green development level $Green_{it}$ of each city. The basis for selecting this indicator is as follows: First, the Urban Health Ecological Index is a comprehensive indicator, including three secondary indicators of ecological economy, ecological society, and ecological environment, which coincide with the connotation of the coordinated development of the economy, society, and the environment as required by green development [16,18,19,20]. Second, the Social Development Research Center of the Chinese Academy of Social Sciences, which has good representativeness and authority in evaluating urban green development, compiles the “China Eco-City Construction and Development Report” also known as the “green book of ecological city”.

In terms of control variables, previous studies have pointed out that the green development of cities is affected by the economic development, industrial structure, and scale of foreign investment in the region [61,62]. Therefore, this paper selects per capita GDP (*pgdp*), the proportion of added value of secondary and tertiary industries (*ind*_2, *ind*_3), and the total number of actual used foreign direct investment (*fdi*) as the control variables of the model. The data are collected from the China City Statistical Yearbook for the corresponding year.

Since the PSM-DID method essentially uses matched variables to regress the low-carbon pilot policy dummies, the multivariate variables are compressed into a single-dimensional propensity score to match the cities of the experimental group and the control group. Therefore, when calculating the propensity score, this paper adds other factors that may have an impact on policy choices as matching variables on the basis of the above control variables, including the average wage of urban employees (*wage*) and the total population at the end of the year (*people*). The data come from the China City Statistical Yearbook for the corresponding year.

In order to eliminate the impact of price changes, the GDP deflator was used to reduce the relevant variables. Table 1 shows the descriptive statistics for each variable.

## 5. Analysis and Test of Empirical Results

### 5.1. Analysis of Benchmark Regression Results

The benchmark regression results for low-carbon pilot policy on the level of urban green development are shown in Table 2. Among them, model (1) to model (4) are the regression results of the ordinary panel model, city fixed effect model, time fixed-effect model and double fixed model, in order. Considering the lag of policy, model (5) is the regression result of the explained variable with one period. Considering the stronger implementation of pilot policies by provincial capital cities and municipalities, resulting in sample selection errors, model (6) reports the regression results after excluding the samples of provincial capital cities and municipalities.

In model (1), the regression coefficient of the interaction term City×Policy is $\beta_{3}=0.0304$, and it passes the significance test with a confidence of 99%. At the same time, model (2) to model (4) with fixed effect is still significantly positive, and the fluctuation range of the regression coefficient is from 0.0282 to 0.0306, indicating that the regression result of $\beta_{3}$ is relatively stable. The above regression results are in line with the expectations of this paper, indicating that the level of green development in pilot cities was improved significantly after the implementation of low-carbon pilot policies compared to non-pilot cities.

The second batch of low-carbon pilot cities was approved in November 2012. In this paper, the policy discontinuity point is set as 2013. However, it takes time for the low-carbon pilot policy to move from the top-level design of the central government to the implementation of the local government and relevant departments. Therefore, the significant policy effects of low-carbon pilot policy may be lagging behind. In model (5), the regression results of explained variables lagging behind for one period show that the regression coefficient of the interaction item is $\beta_{3}=0.0164$. Although the regression coefficient decreases, it still passes the significance test. This conclusion means that the pilot policy plays a continuous role in promoting urban green development, but the effect of the low-carbon pilot policy decreases with the passage of time.

Compared with other cities, the implementation of the low-carbon pilot policy by provincial capital cities and municipalities is generally subject to more stringent supervision; at the same time, provincial capital cities and municipalities can often have access to richer economic resources and policy preferences. Based on this, this paper judges that provincial capital cities and municipalities have stronger enforcement of low-carbon pilot policies. Model (6) reports the regression results after excluding the samples of provincial capital cities and municipalities. The regression coefficient of the interaction term is still significantly positive at $\beta_{3}=0.0256$, but compared with the regression results of model (1) to model (4) under the full sample, its significance level and regression coefficient size decreased. The above regression results show the following: First, excluding the sample of provincial capital cities and municipalities does not change the core research conclusion of this paper, that is, the low-carbon pilot policy can significantly improve the level of green development in pilot cities. Second, the implementation of the low-carbon pilot policy by local governments is directly related to the policy effect of the low-carbon pilot; the stronger the implementation, the more obvious the policy effect. In the next part of the heterogeneity analysis, this paper will distinguish the eastern, central, and western cities, and further elaborate the relationship between the implementation strength and the effect of the low-carbon pilot policy.

Taking the regression results of the double fixed model (4) as an example, the regression coefficient of the urban dummy variable City is also significantly positive ($\beta_{1}=0.1895$), indicating that the green development level of pilot cities is significantly better than that of non-pilot cities. However, the regression coefficient of the dummy variable of the policy break point  Policy is significantly negative ($\beta_{2}=-0.1028$), which means that the green development level of each city declined after 2013 compared with that before the implementation of the low-carbon pilot policy. This conclusion does not seem to meet the expectations of this paper, but it confirms the actual situation. This article chooses the urban health ecological index to measure the green development level of each city, which is different from the single carbon emission index used in other literature. The urban health ecological index is an investigation of the overall quality of the coordinated development of the city’s “economy–society–environment” in the three aspects of ecological economy, ecological society, and ecological environment. After the 18th CPC National Congress, China’s economic development entered the stage of “three-phase superposition” of the shifting period of growth rate, the painful period of structural adjustment, and the digestion period of early stimulus policies. Therefore, the structural decline in the rate of economic growth has a certain impact on the healthy ecological index of cities.

In terms of control variables, the regression results of the double fixed model (4) show that the per capita GDP has a negative but not significant impact on the urban green development level. Combined with the research conclusions of the existing literature, this paper suggests that China is in the turning stage of the Environmental Kuznets Curve (a curve describing the inverted U-shaped relationship between environmental pollution and economic development) [63,64]. On the one hand, the extensive mode of urban economic development is gradually changing. Meanwhile, the public awareness of environmental protection is gradually expanding. In the industrial structure, the proportion of secondary industries has a negative but not significant impact on the urban green development level, while the proportion of tertiary industries significantly limits the improvement of the urban green development level. The possible reason is that the development of tertiary industries is not as easy as people think, e.g., the rapidly expanding transportation and logistics sectors in recent years. At the same time, the impact of foreign direct investment on the level of urban green development is not significant, which is in line with the inference of the “pollution shelter” hypothesis; that is, although foreign direct investment reduces the environmental quality of the urban health ecological index, it has a positive impact on urban economic development.

### 5.2. Robustness Test

(1) Parallel trend test. The premise condition of common trend needs to be satisfied before adopting the DID method for analysis; specifically, without low-carbon pilot policy interference, the green development level for the experimental and control groups should have the same development trend; otherwise, the control group cities cannot be used to control the impact of time effect on the experimental group cities.

**Figure 3:**
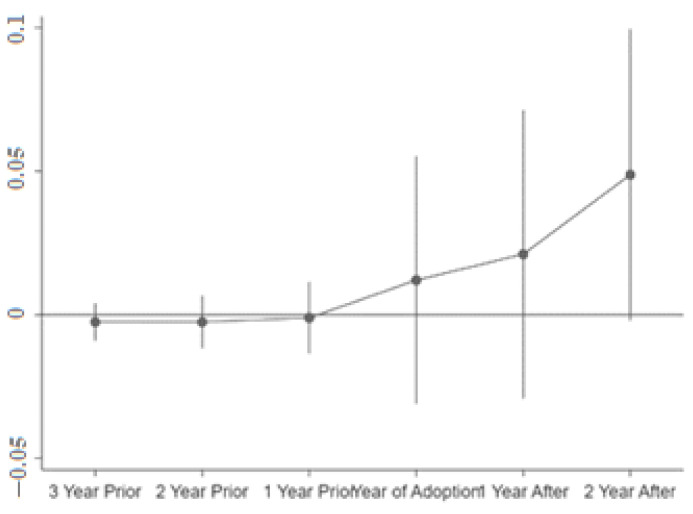
Parallel trend test results.

In order to test the parallel trend hypothesis, this paper selects three years before and after the implementation of the low-carbon pilot policy, namely 2010–2015, to generate the interaction term between the year virtual variable and the experimental group virtual variable, and then regresses the interaction term as the explanatory variable and uses the regression coefficient of the interaction item to draw the coefficient fluctuation diagram as shown in Figure 3. From Figure 3, it is intuitive to see that before the implementation of the low-carbon pilot policy, the regression coefficient of the interaction term fluctuates around 0; after the implementation of the low-carbon pilot policy, the regression coefficient of the interaction term is significantly larger than 0. The aforementioned findings demonstrate that the research in this paper has passed the parallel trend hypothesis test, meaning that the control group cities can be used to eliminate the effect of time on the experimental group cities in order to extract the policy effect of the low-carbon pilot on the experimental group cities.

(2) Counterfactual test. Theoretically, during this study period, in addition to the implementation of low-carbon pilot policy having an impact on urban green development, the promulgation of other policies and the perturbation of stochastic factors may cause changes in the level of urban green development. To eliminate interference from other policies and stochastic factors, this paper performs a counterfactual test by changing the implementation discontinuity point of the low-carbon pilot policy. Specifically, the implementation of the low-carbon pilot policy is brought forward by one year. At this time, if the regression coefficient $\beta_{3}$ of the interaction item City×Policy is still significant, it indicates that the difference of green development level between the experimental group and the control group may be caused by other policies or random factors. If the regression coefficient of the interaction term is not significant, this verifies that the difference in the level of green development between the two types of cities is caused by the impact of the low-carbon pilot policy.

The regression results of the counterfactual test are shown in Table 3. Model (7) and model (8) are the counterfactual test results of assuming that the implementation time of the low-carbon pilot policy advanced two years to 2011; the provincial capital cities and municipalities are excluded from model (8). Model (9) and model (10) are the counterfactual test results of assuming that the policy implementation time advanced three years to 2010; the provincial capital cities and municipalities are also excluded from model (10). The regression results of each model show that the regression coefficient $\beta_{3}$ of City×Policy fail to pass the significance test, which verifies that the difference of green development level between pilot cities and non-pilot cities is caused by the impact of low-carbon pilot policy, and the interference of other policies or random factors has no significant impact on the difference of green development level between the two types of cities.

(3) Sample expansion test. In the benchmark regression model, the second batch of low-carbon pilot cities with a certain size and relatively mature development is selected as the research object. In order to further verify whether the conclusions of the benchmark regression model are robust, this paper brings the first batch of eight low-carbon pilot cities into the empirical analysis framework and sets the discontinuities of the first batch and the second batch of low-carbon pilot policy as 2011 and 2013, respectively (the China Eco-City Construction and Development Report after 2016 changed the evaluation index system for the calculation of urban health ecological index. In order to ensure consistency of the data calibrations and eliminate the errors caused by the changes of evaluation criteria, the third batch of low-carbon pilot cities were not included in the analysis framework).

Table 4 reports the regression results after extending the study sample to the first and second batch of low-carbon pilot cities. Among them, model (11) to model (13) show the regression results of the whole sample and the explained variable lag for one period; the samples of provincial capital cities and municipalities are excluded. It can be seen from Table 4 that the regression coefficient $\beta_{3}$ of City×Policy is significantly positive, which is consistent with the conclusions obtained in the benchmark regression model, which further verifies that the low-carbon pilot project has a significant policy effect on promoting urban green development.

### 5.3. Heterogeneity Analysis of Policy Effect

In order to implement the central government’s top-level design for the construction of low-carbon pilot cities, each pilot area formulated specific action plans. However, different cities have different economic bases, which inevitably affect the implementation of the low-carbon pilot policy. Generally speaking, cities in eastern China have stronger economic foundations than those in central and western regions, and they are more motivated to promote the construction of low-carbon pilot projects. Therefore, it is necessary to investigate whether low-carbon pilot policies show cross-regional heterogeneity in promoting urban green development.

On the basis of formula (1), the regional dummy variables *Area1* and *Area2* are included in this paper.

$$Area1=\left\{\begin{array}{c} 1,\;\mathrm{Eastern}\;\mathrm{cities}\;\\ 0,\;\mathrm{Non}-\mathrm{eastern}\;\mathrm{cities} \end{array}\right.Area2=\left\{\begin{array}{c} 1,\;\mathrm{Western}\;\mathrm{cities}\;\\ 0,\;\mathrm{Non}-\mathrm{western}\;\mathrm{cities} \end{array}\right.$$

By multiplying the regional dummy variable with the urban dummy variable and the dummy variable of the policy implementation discontinuity point, the model shown in Formula (2) is obtained, in which the regression coefficient $\beta_{3}$ reflects the cross-regional heterogeneity of the effect of the low-carbon pilot policy.

(2) $$Green_{it}=\beta_{1}City_{i}+\beta_{2}Policy_{t}+\beta_{3}City_{i}\times Policy_{t}\times Area+\lambda X_{it}+\nu_{i}+\mu_{t}+\varepsilon_{it}$$

Table 5 reports the regression results for Equation (2). Among them, model (14) to model (16) and model (17) to model (19) are the regression results of the whole sample, the explained variable lag for one period, and the sample excluding provincial capital cities and municipalities after the regional dummy variable *Area*1 and *Area*2 are included.

First of all, for the regression results of model (14) to model (16), the regression coefficient $\beta_{3}$ of City×Policy×Area is significantly positive, indicating that the low-carbon pilot policy played a more significant role in promoting the green development of cities in the eastern region. In view of the stronger economic foundation of cities in the eastern region, this paper argues that the economic foundation plays a positive role in the process of promoting the green development of cities through low-carbon pilot policies. Secondly, in terms of the regression results of model (17) to model (19), the regression coefficient $\beta_{3}$ of City×Policy×Area either fails the significance test or is significantly negative, which means that in the western region, the low-carbon pilot policy has not played a positive role in promoting the green development of cities and has even hindered the promotion of green development in the western region. Analyzing the reasons, this paper holds that ① the economic foundation of cities in the western region is relatively weak, which restricts the implementation of the low-carbon pilot policy in western cities; ② In the process of industrial transformation and upgrading in the eastern region, the cities in the western region have undertaken the important task of absorbing the transfer of heavy industry from the eastern region. The low-carbon pilot policy, which focuses on the establishment of a low-carbon industrial system characterized by high-tech content, low pollution emissions, and low energy consumption, does not fit well with the urban development in western China.

## 6. Conclusions

In this paper, we use the PSM-DID model to analyze and test the theoretical mechanism and policy effects of low-carbon pilot cities to promote urban green development, taking the second batch of low-carbon pilot cities released by the NDRC as the research object. The research conclusion points out that compared with non-pilot cities, the low-carbon pilot policy has promoted the coordinated green development of low-carbon pilot cities in terms of economy, society, and environment from the three aspects of constructing a low-carbon industrial system to improve the green connotation of urban economic development, advocating a low-carbon lifestyle to promote green progress in urban social life, and establishing low-carbon assessment constraints to force improvement in urban environmental governance. The empirical analysis and robustness test with the second batch of low-carbon pilot cities as the sample also confirmed the significant role of the low-carbon pilot policy in promoting the green development level of pilot cities, but also show that there is significant cross-regional heterogeneity in the policy effect of low-carbon pilot cities. Specifically, the low-carbon pilot policy has a more significant policy effect in promoting the green development of cities in the eastern region, but it has not achieved the expected policy effect in the cities of the western region and even hinders the promotion of the green development level of the cities in this area. The research presented in this paper is important for shedding light on the theoretical mechanisms of low-carbon pilot policies to promote urban green development and to scientifically evaluate the policy effects. Based on the above research conclusions, this paper considers the construction of low-carbon cities as a breakthrough and puts forward the following countermeasures and suggestions to promote the coordinated green development of the urban economy, society and environment.

First, we the experience of low-carbon pilot projects should be established in order to allow for the expansion to non-pilot cities. The conclusion of this paper validates the positive role of low-carbon pilot cities in promoting urban green development and points out that compared with non-pilot cities, the level of green development of low-carbon pilot cities significantly improved after the implementation of the pilot policy. Though China is still experiencing challenges in terms of energy conservation and emission reduction, the expectations for sustainable urban development are increasingly strong against the backdrop of new urbanization. Therefore, this paper suggests that the beneficial experiences of low-carbon pilot cities should be catalogued in a timely manner, especially the effective measures in building low-carbon industrial systems, advocating low-carbon lifestyles, and establishing low-carbon assessment constraints, in order to promote expansion to non-pilot areas. In the process, it will also be necessary to integrate the specific economic characteristics and social foundations of each city to carry out low-carbon city construction according to local conditions and urban policies.

Second, we should improve the implementation of local government policies and pay attention to the formulation of construction plans that are in line with the actual situation of the city. The conclusion of this paper also points out that, compared with cities in central and western regions, low-carbon pilot projects have achieved more significant policy effects in provincial capital cities, municipalities, and eastern cities. Although there are complex factors behind this phenomenon, the improved implementation brought about by a good economic foundation and strict administrative supervision is undoubtedly one of the most important factors. Therefore, this paper suggests that local governments should, on the one hand, improve the implementation of a national low-carbon pilot policy and put an end to bureaucratization. On the other hand, it is also necessary to formulate and implement scientific, orderly and targeted low-carbon city construction plans and objectives, taking into account the actual economic foundation of each city and the medium and long-term development plans in the field of urban economic and social development.

Third, we should pay attention to the cultivation of the endogenous driving force of urban green development and promote its decoupling from the pilot policy. The value of a pilot policy is reflected in its experimental properties and in its policy properties, that is, in the process of urban green development transformation, policy incentives are supplemented to reduce obstacles and difficulties in the transformation process. However, urban green development cannot continuously rely on policy incentives to move forward, as the city’s green development will face retrogression once the pilot policy is cancelled. Therefore, we must focus on nurturing the endogenous driving force of urban green development and promoting its decoupling from pilot policies in a timely manner. For example, enterprises need to find new economic growth points from the transformation of the production modes to limit dependence on extensive production. Residents need to cultivate low-carbon living as well. From this point of view, the low-carbon construction plan or scheme of each city cannot be limited to policy incentives, but also needs to tap into the potential value of low-carbon production modes and create a healthy low-carbon environment.

Fourth, the endogenous driving force of urban green development is also inseparable from the participation of residents [65,66]. Public participation plays an important role in improving policy implementation, enhancing social cohesion, and promoting rational decision making [67,68]. Social tipping interventions have been suggested as a policy tool to bring about this change [59,69]. Social tipping means that a small number of people committed to the target behavior can create a self-reinforcing drive to establish the target behavior as a social norm [70]. In addition to administrative supervision, the public also enables social supervision of the pilot policy, which increases the illegality of polluting enterprises and ensures that the construction of low-carbon cities brings about the maximum ecological benefits [71,72]. At the same time, once residents’ ecological values are cultivated, the power of the public will continue to push the city toward green development even if the pilot policies of low-carbon cities are cancelled.

## Figures and Tables

**Table 1:** Descriptive statistics.

Variable	Full Sample			Experimental Group			Control Sample		
	Average Value	Standard Deviation	Sample Size	Average Value	Standard Deviation	Sample Size	Average Value	Standard Deviation	Sample Size
ln*Green*	−0.2333	0.0788	672	−0.2116	0.0706	120	−0.2381	0.0797	552
ln*pgdp*	10.2166	0.5968	672	10.4452	0.5894	120	10.1669	0.5872	552
ln*ind_2*	3.8879	0.2616	672	3.7830	0.2566	120	3.9107	0.2573	552
ln*ind_3*	3.6608	0.3091	672	3.8138	0.3240	120	3.6275	0.2958	552
ln*fdi*	10.6998	1.5049	672	11.6719	1.7026	120	10.4885	1.3716	552
ln*wage*	10.6017	0.3311	672	10.7463	0.3673	120	10.5702	0.3143	552
ln*people*	5.8941	0.7736	672	6.2212	0.8257	120	5.8230	0.7437	552

**Table 2:** Regression results of benchmark model.

Model	Model (1)	Model (2)	Model (3)	Model (4)	Model (5)	Model (6)
City	−0.0048 (−0.45)	0.2476 *** (5.74)	−0.0089 (−0.86)	0.1895 *** (3.85)	0.1954 *** (4.61)	0.1358 *** (3.79)
Policy	−0.1026 *** (−19.34)	−0.0685 *** (−9.86)	−0.1490 *** (−16.37)	−0.1028 *** (−7.67)	−0.0562 *** (−5.61)	−0.1121 *** (−5.94)
City×Policy	0.0304 *** (2.67)	0.0282 ** (2.56)	0.0306 *** (2.81)	0.0284 *** (2.65)	0.0164 * (1.68)	0.0256 * (1.67)
*pgdp*	0.0396 *** (5.87)	−0.0138 (−1.14)	0.0460 *** (6.84)	−0.0078 (−0.55)	−0.0244 ** (−2.06)	−0.0343 (−1.49)
*ind*_2	−0.0273 (−1.21)	−0.0048 (−0.11)	−0.0391 * (−1.78)	−0.0580 (−1.35)	0.0071 (0.18)	−0.0479 (−0.92)
*ind*_3	0.0080 (0.41)	−0.0823 ** (−2.58)	0.0126 (0.67)	−0.0613* (−1.93)	−0.0310 (−1.08)	−0.0198 (−0.55)
*fdi*	0.0033 (1.18)	−0.0099 ** (−1.99)	0.003 2 (1.18)	−0.0082 (−1.57)	−0.0034 (−0.74)	−0.0139** (−2.13)
*City fixed effect*	no	yes	no	yes	yes	yes
*Time fixed effect*	no	no	yes	yes	yes	yes
*R*-*squared*	0.3591	0.4047	0.4159	0.4382	0.4098	0.4889
*Sample size*	672	672	672	672	588	488

Note: The figures in parentheses are z-values; ***, **, and * indicate significance at the 99%, 95%, and 90% confidence levels, respectively.

**Table 3:** Counterfactual test results.

Model	Model (7)	Model (8)	Model (9)	Model (10)
City	0.1876 *** (3.68)	0.1417 *** (3.64)	0.1869 *** (3.73)	0.1386 *** (3.71)
Policy	−0.1002 *** (−7.34)	−0.1088 *** (−5.66)	−0.1009 *** (−7.46)	−0.1099 *** (−5.77)
City×Policy	0.0126 (1.04)	0.0052 (0.30)	0.0164 (1.52)	0.0103 (0.67)
*pgdp*	−0.0073 (−0.51)	−0.0344 (−1.48)	−0.0071 (−0.50)	−0.0339 (−1.46)
*ind*_2	−0.0634 (−1.47)	−0.0439 (−0.84)	−0.0621 (−1.44)	−0.0454 (−0.87)
*ind*_3	−0.0670 ** (−2.10)	−0.0120 (−0.55)	−0.0661 ** (−2.07)	−0.0204 (−0.56)
*fdi*	−0.0075 (−1.43)	−0.0140 ** (−2.11)	−0.0076 (−1.45)	−0.0138 ** (−2.10)
*City fixed effect*	yes	yes	yes	yes
*Time fixed effect*	yes	yes	yes	yes
*R-squared*	0.4324	0.4856	0.4336	0.4860
*Sample size*	672	488	672	488

Note: The figures in parentheses are z-values; *** and ** indicate significance at the 99% and 95% confidence levels, respectively.

**Table 4:** Sample expansion test results.

Model	Model (11)	Model (12)	Model (13)
City	0.1646 *** (3.57)	0.1803 *** (4.52)	0.1270 *** (3.69)
Policy	−0.0419 ** (−2.55)	−0.0229 * (−1.76)	−0.0334 (−1.19)
City×Policy	0.0345 *** (3.69)	0.0166 ** (2.14)	0.0262 * (1.88)
*pgdp*	−0.0033 (−0.26)	−0.0230 ** (−2.15)	−0.0258 (−1.26)
*ind*_2	−0.0789 * (−1.88)	−0.0092 (−0.24)	−0.0723 (−1.40)
*ind*_3	−0.0635 ** (−2.02)	−0.0355 (−1.26)	−0.0249 (−0.69)
*fdi*	−0.0070 (−1.38)	−0.0024 (−0.54)	−0.0127 * (−1.94)
*City fixed effect*	yes	yes	yes
*Time fixed effect*	yes	yes	yes
*R-squared*	0.4215	0.3974	0.4805
*Sample size*	736	644	512

Note: The figures in parentheses are z-values; ***, **, and * indicate significance at 99%, 95%, and 90% confidence levels, respectively.

**Table 5:** Cross-regional heterogeneity test of low-carbon pilot policy promoting urban green development.

Model	Model (14)	Model (15)	Model (16)	Model (17)	Model (18)	Model (19)
	*Area*1			*Area*2		
City	0.1817 *** (3.73)	0.1868 *** (4.45)	0.1096 *** (3.14)	0.2035 *** (4.82)	0.2031 *** (4.81)	0.1489 *** (4.22)
Policy	−0.1041 *** (−7.89)	−0.0587 *** (−5.97)	−0.1174 *** (−6.45)	−0.0524 *** (−5.36)	−0.0527 *** (−5.38)	−0.1045 *** (−5.59)
City×Policy×Area	0.0576 *** (4.34)	0.0418 *** (3.47)	0.0987 *** (5.36)	0.0048 (0.30)	0.0089 (0.50)	−0.0583 ** (−1.97)
*pgdp*	−0.0078 (−0.56)	−0.0227 * (−1.94)	−0.0364 (−1.63)	−0.0266 ** (−2.25)	−0.0262 ** (−2.22)	−0.0378 (−1.64)
*ind*_2	−0.0411 (−0.96)	0.0153 (0.39)	−0.0251 (−0.50)	0.0044 (0.11)	0.0046 (0.12)	−0.0265 (−0.50)
*ind*_3	−0.0486 (−1.53)	−0.0207 (−0.72)	0.0015 (0.04)	−0.0355 (−1.23)	−0.0360 (−1.25)	−0.0075 (−0.20)
*fdi*	−0.0084 (−1.64)	−0.0041 (−0.91)	−0.0149 ** (−2.35)	−0.0030 (−0.65)	−0.0029 (−0.64)	−0.0152 ** (−2.32)
*City fixed effect*	yes	yes	yes	yes	yes	yes
*Time fixed effect*	yes	yes	yes	yes	yes	yes
*R-squared*	0.4494	0.4205	0.518 8	0.4065	0.4067	0.4903
*Sample size*	672	588	488	672	588	488

Note: The figures in parentheses are z-values; ***, **, and * indicate significance at 99%, 95%, and 90% confidence levels, respectively.

## Data Availability

Datasets and materials used in this study are available upon request to the authors.
